# Estrogen Replacement in Young Hypogonadal Women—Transferrable Lessons From the Literature Related to the Care of Young Women With Premature Ovarian Failure and Transgender Women

**DOI:** 10.3389/fendo.2019.00685

**Published:** 2019-10-11

**Authors:** Du Soon Swee, Usman Javaid, Richard Quinton

**Affiliations:** ^1^Department of Endocrinology, Royal Victoria Infirmary, Newcastle upon Tyne, United Kingdom; ^2^Department of Endocrinology, Singapore General Hospital, Singapore, Singapore; ^3^Institute of Genetic Medicine, Newcastle University, Newcastle upon Tyne, United Kingdom

**Keywords:** hormonal replacement therapy, estrogen (17b-estradiol), conjugated estrogen, ethinyl estradiol (EE), female hypogonadism, premature ovarian failure, transgender, cross-sex hormonal treatment

## Introduction

Nearly two decades have passed since the release of Women's Health Initiative (WHI) postmenopausal hormone therapy trial findings, yet the medical community, and general public remain unsettled by ongoing debate over the benefits and safety of sex hormone replacement therapy (HRT). Among the contentious issues is the elevated risk of venous thromboembolism (VTE) and stroke observed in HRT users (1). While major guidelines rightly recommend the use of transdermal estradiol in women with risk factors, little attention has been given to the potential impact of the type of estrogen molecule. This review aims to highlight the importance of selecting appropriate estrogen therapy to enhance safety.

## Misinterpretation of WHI Data Compromises Care of Hypogonadal Women

Hypogonadism in women of pre-menopausal age group is more frequent than is commonly anticipated; spontaneous or autoimmune primary ovarian insufficiency affects ~1% of the female population, and an estimated 5% experience early menopause prior to age 45 (2). Other important causes of premature estrogen deficiency include congenital conditions such as Turner syndrome and Kallmann syndrome, as well as surgical oophorectomy. For these patients, HRT is a well-established endocrine treatment aimed to replace estrogen physiologically until at least the average age of menopause. Untreated individuals are at substantial risk of sexual dysfunction, genitourinary symptoms, accelerated bone loss, vasomotor symptoms, and coronary heart disease (CHD) (3).

Similarly, in the management of postmenopausal women suffering from climacteric syndrome, estrogen is unequivocally more efficacious compared to non-estrogen-based pharmacological treatments, and plays a crucial role in holistic menopause management particularly in those with impaired quality of life from persistent vasomotor symptoms.

Unfortunately, many healthcare providers and patients became resistant to the use of HRT in the aftermath of WHI. Not only are menopausal women in their 50s and those with vasomotor symptoms unnecessarily deprived of HRT (4), there are also worrying signs of under-treatment among young hypogonadal women; a recent Swedish report on women with central hypogonadism found that at least half of the cohort failed to receive adequate replacement during their estrogen-deficient premenopausal years, placing them at heightened risk of complications in the ensuing years (5).

Prior to the landmark WHI trial, several large-scale observational studies were actually in favor of HRT's protective effects, as treated women were found to be at lower risk of CHD and mortality (6). To substantiate these observations, WHI postmenopausal hormone trials set out to investigate HRT in women aged 50–79 years; participants randomized to intervention arms received either conjugated equine estrogen (CEE) 0.625 mg alone (absent uterus) or with cyclical medroxyprogesterone acetate (MPA) 2.5 mg (intact uterus). Not only did WHI unexpectedly fail to demonstrate cardiovascular benefits, a disconcerting increase in incidence of breast cancer, stroke and VTE in treatment arms led to the premature closure of study after a median follow-up of 5.6 years (7).

Around the same time, the Heart and Estrogen/Progestin Replacement Study (HERS) trial also reported neutral effect of HRT (CEE+MPA vs. placebo) on CHD risk along with increased VTE events (8). Since then, the “timing hypothesis” has been widely proposed to explain the discordance in observational and trial findings because, unlike in typical clinical settings where most patients considered for HRT are early post-menopausal, the average age at which WHI subjects were initiated on HRT was 63.3 years (9). Indeed, *post-hoc* analyses showed better outcomes including reduction in CHD risk in subgroups of age <60 or <10 years from the time of menopause (10), with corroborative CT evidence of lower coronary calcified-plaque burden compared to placebo arm (11).

Aside from age factor, the type of estrogen therapy should also be carefully considered in HRT decision-making. It is imperative that estrogen products with the greatest safety margins be selected. However, this is an aspect that has often been overlooked in HRT guidance, with results of WHI/HERS often being inappropriately applied to all estrogen formulations. As will be elaborated further, non-physiological estrogenic compounds—by virtue of having greater propensity in inducing prothrombogenic state across ages—should be avoided in patients prescribed HRT.

## Choice of Estrogen Formulation Influences Treatment Risks and Benefits

There are three main types of estrogen formulations available for therapeutic purposes, namely 17β-estradiol (E_2_), ethinylestradiol (EE) and CEE. The former (available in oral and transdermal formulations) is the predominant endogenous human estrogen. To overcome its poor oral bioavailability (<10%), E_2_ is typically esterified or micronized; pro-drug esters, such as estradiol valerate and estradiol acetate, undergo hydrolysis rapidly following absorption to release E_2_ into the systemic circulation, while the microcrystalline structure of micronised estradiol (principally as estradiol hemihydrate) facilitates accelerated absorption by its larger compound surface area and thus minimizing first-pass metabolism (12). Conversely, transdermal application of E_2_, which has moderate skin permeability, avoids first-pass effect, and hence generates an E_2_:E_1_ (estrone, a metabolite of E_2_) profile similar to normal physiology, whereas E_1_ concentrations are higher after oral E_2_ administration (13). However, the weak potency of E_1_ does not have significant impact on the overall estrogenic bioactivity (14).

In contrast, CEE and EE are non-physiological because of their different molecular structure and properties. EE—a near-universal component of combined oral contraceptives (COCs)—is a potent synthetic E_2_ analog with a 17α-ethinyl substitution that binds to estrogen receptors α and β with high affinity, prevents the oxidation of the 17β-hydroxy group, as well as irreversibly inhibits CYP enzymes involved in the metabolism of steroids, resulting in a very reactive intermediate (12). CEEs are urine derivatives from pregnant horses and is a complex mix of numerous estrogenic compounds with varying receptor affinity, pharmacokinetics and biologic potency, as well as other non-estrogenic steroids with unknown effects (12). Additionally, both EE and CEE have considerably greater hepatic stimulatory effect, altering the synthesis of various proteins including angiotensinogen, SHBG and coagulation factors.

Given these fundamental pharmacological differences, biological effects are expected to be dissimilar, and hence the adverse effects observed in older trials employing non-physiological estrogen would not be generalisable to all estrogen formulations. Indeed, emerging data are demonstrating comparatively greater safety and efficacy associated with E_2_ use.

In a population-based, case-control study of ~400 postmenopausal women aged 30–79 years using oral hormone therapy, CEE use was significantly associated with increased venous thrombosis risk (odds ratio 2.08) and a trend toward increased myocardial infarction (MI) risk when compared with E_2_ (15). Further investigations demonstrated a higher endogenous thrombin potential-based normalized activated protein C (APC) sensitivity ratio as one of the mechanisms for the elevated clotting propensity observed in CEE users. This is in line with a recent large UK observational study of general female population aged 40–79 which found that among oral HRT, CEE(+MPA) had the greatest risk while E_2_(+dydrogesterone) had the lowest risk (16). Likewise, Danish Osteoporosis Prevention Study showed no evidence of increased thrombotic or stroke risk in women with recent menopause onset who received E_2_(± norethisterone) replacement and followed for up to 16 years (17). Moreover, a significant reduction in combined end-point of mortality and hospitalisations for congestive heart failure or MI was demonstrated.

Similarly, recent HRT intervention trials in younger women with premature ovarian failure have reported encouraging data with E_2_ therapy (Table 1). Improvement in BMD, particularly at lumbar spine, was consistently observed across studies, with E_2_ demonstrating superiority to COCs (19, 21). Furthermore, E_2_ has beneficial effects on several cardiovascular and uterine parameters, which could have far-reaching impact on long-term cardiovascular health and possibly fertility treatment outcomes, respectively (20, 22). These studies also provided evidence for dose titration to achieve physiological serum E_2_ levels (21, 23). More importantly, both oral and transdermal E_2_ therapy were safe and well-tolerated in these trials.

**Table 1:** Summary of hormone therapy studies in **(A)** young women with premature ovarian failure (recent HRT trials), and **(B)** transgender females (retrospective & cross-sectional studies).

**References**	**Study design**	**HRT regimen**	**Subjects**	**Key findings**	**Remarks**
**(A)**					
Popat et al. (18)	3-year prospective, randomized, double-blind, single-center, placebo-controlled clinical trial.	Estradiol patch 100 μg/d & cyclical oral MPA 10 mg/d for 12 d/mo, ± Testosterone (T) patch 150 μg/d.	145 women with spontaneous 46, XX primary ovarian insufficiency vs. 70 healthy female controls.	Normalization of bone mineral density (BMD): ↑2.45% at neck of femur (NoF), ↑7.7% at lumbar spine (LS).Increase in bone formation markers.	Transdermal T did not provide additional benefit. 5 subjects (4 received T) had skin irritation, redness, hirsutism, & oily skin.
Cartwright et al. (19)	Open-label randomized trial comparing effects of HRT and combined oral contraceptive pill (COCP) on bone density.	HRT (Estradiol 2 mg/d & levonorgestrel 75 μg for 12d/mo) vs. COCP (EE 30 μg & levonorgestrel 150 μ for 21d/mo followed by 7-day break).	50 women with spontaneous POI recruited, of whom 30 elected for estrogen therapy (HRT = 15, COCP = 15). 36 completed the trial (no treatment 52%; HRT 60%; COCP 80%).	HRT significantly ↑ BMD at LS at 2 years, compared to COCP, while NoF and total hip BMD remained stable in all treated subjects. BMD decreased at all sites in untreated women.	HRT is superior to COCP in improving bone density at LS. No adverse cardiovascular events reported.
University of Edinburgh group (20–22)	12-month open-label randomized controlled crossover trial, comparing effects of physiological HRT vs. COCP on:	Estradiol patch (100 μg/d for week 1, 150 μg/d for weeks 2–4) & vaginal progesterone (200 mg/12 h for weeks 3–4) vs. COCP (EE 30 μg/d & norethisterone 1.5 mg/d for weeks 1–3 followed by 7 “pill-free” days).	34 women with primary ovarian failure (POF) attributed to chemotherapy or radiotherapy, idiopathic or surgical treatment, or Turner syndrome.		Only 4 clearly withdrew because of intolerance to the treatment, which was adverse reaction to the patch adhesives.
O'Donnell et al. (20)	Uterine health		17 women completed study; data from 25 subjects were analyzed.	Significant beneficial effect on endometrial thickness, and trend toward greater uterine volume.	HRT could benefit women with POF seeking infertility treatment by improving uterine physical characteristics.
Crofton et al. (21)	Skeletal health		18 women completed study.	Significant improvement in LS BMD *z*-scores observed in HRT but not COCP group, & only HRT was associated with an increase in bone formation markers.	The positive correlation of HRT's beneficial effect on LS BMD to E_2_ levels underscores the importance of ensuring treatment adequacy.
Langrish et al. (22)	Cardio-vascular health		18 women completed the study.	HRT was associated with lower mean 24-h systolic & diastolic BP throughout the treatment period, along with reduced plasma angiotensin II and serum creatinine.	Compared to COCP, physiological HRT could have beneficial long-term cardiovascular health benefits.
Torres-Santiago et al. (23)	12-month randomized clinical trial to assess the metabolic effects of oral vs. transdermal E_2_.	Cyclical estradiol (oral or transdermal) for weeks 1–3, with doses titrated to normal E_2_ range in both groups, & MPA 10 mg from days 14–21 each month.	40 women with Turner syndrome; 20 in each treatment arm.	No significant difference in body composition, lipid oxidation, and lipid concentrations.	Oral and transdermal E_2_ exert similar metabolic effects when titrated to normal E_2_ range. No adverse event reported.
**(B)**					
Asscheman et al. (24)	Observational cohort study	Various estrogen regimens	966 Transgender females; mean age at therapy initiation was 31.4 ± 11.4 years; median follow-up of 18.5 years.	Current EE use was associated with 3-fold increase in risk of cardiovascular mortality.	No increased risk was observed in former EE users who had changed to other formulations & lower doses of E_2._
Dittrich et al. (25)	Retrospective cohort study	Monthly injections of gonadotrophin-releasing hormone agonist, & oral estradiol valerate 6 mg/d for 2 years.	60 transgender females, mean age 38.3 ± 11.3 years, treated with	One venous thrombosis occurred in a 62-year-old patient with known homozygous methylenetetrahydrofolate reductase mutation (genetic predisposition to thrombosis).	Increase in LS and NoF BMD observed. Overall safe and effective treatment.
Ott et al. (26)	Retrospective cohort study	Transdermal E_2_ (2 ×100 μg/week); + oral cyproterone acetate & finasteride if yet to undergo sex reassignment surgery (SRS).	162 transwomen, mean age 36.6 ± 10.9 years, mean follow up period of 64.2 ± 38.0 months.	None developed VTE under cross-sex hormone therapy.	Notably 8.0% of subjects had thrombophilic defect (activated protein C resistance).
Arnold et al. (27)	Retrospective chart review	Oral estradiol therapy (4–8 mg/d & spironolactone pre-SRS, 2–4 mg/d only post-SRS).	676 transwomen, mean age 33.2 ± 10.8 years, treated for a mean of 1.9 years.	1 case of VTE; incidence of 7.8 events per 10,000 person-years.	Subject was in her 20s with severe obesity (BMI of 37 kg/m^2^).
Getahun et al. (28)	Electronic medical record-based cohort study	All types of estrogens, with subgroup analyses of “only estradiol or estradiol first” (E_2_ group), & “only non-estradiol or non-estradiol first” (non-E_2_ group) within the estrogen initiation cohort.	2,842 transwomen, including 853 in estrogen initiation cohort. Mean follow-up of 4.0 years.	Adjusted hazard ratio (HR) for ischaemic stroke were 25.4, 2.8, and 1.8 in non-E_2_ group, E_2_ group, and overall cohort, respectively.Adjusted HR for VTE were 2.8 and 2.1 in E_2_ group and overall cohort, respectively. Not calculated for non-E_2_ group due to small numbers.	Although detailed comparison of risks between various estrogen formulations is not possible from limited data, it is notable that non-E_2_ group had a 9-fold higher risk than E_2_-group for ischemic stroke.
Seal et al. (29)	Controlled, retrospective case audit	Various types of estrogen formulations.	165 transgender women, mean age 45.7 ± 10.0 year, with a mean follow-up of 8.95 ± 4.87 years.	VTE occurred in 1.2%, more frequently in those treated with CEE vs. estradiol valerate.	Nearly 8-fold increased VTE risk with CEE use compared to estradiol valerate.
van Kesteren et al. (30)	Retrospective, descriptive study	EE 100 μg/d & cyproterone acetate 100 mg/d; in subjects age >40 years, transdermal E_2_ was preferred (since 1989).	816 transwomen, mean age 41, treated for 7,734 patient-years.	36 cases of VTE were attributed to hormone therapy; all but one were oral EE users.	The switch to transdermal E_2_ in age >40 years nearly ameliorated VTE risk.
Asscheman et al. (31)	Retrospective medical chart review	EE 100 μg/d and cyproterone acetate 100 mg/d.	303 transwomen, treated for 1,333 patient-years.	19 cases (6.3%) of VTE	EE-based therapy was associated with 45-fold increased risk of VTE. Higher risk was also found in age >40 years.
Wierckx et al. (32)	Multicentre 1-year prospective study	Transwomen <45 years received estradiol valerate 4 mg/d whereas those >45 years received transdermal E2 100 μ/d. All had cyproterone 50 mg/d.	Ghent: 47 subjects, mean age 31.7 ± 14.8; Oslo: 6 subjects, mean age 19.3 ± 2.4.	No cardiovascular or VTE events.	Low risk for adverse events at 1-year follow-up, which is significant considering earlier reports of high incidence of VTE during the first year of cross-sex hormone therapy.
Wierckx et al. (33)	Cross-sectional study	Various estrogen formulations	214 transwomen on average treatment period of 7.4 years.	5% had VTE; half occurred in the first year of therapy; Only 2 subjects were on EE or CEE, whilst at least 3 were using transdermal E_2_ at the time of incident.	Findings deviate from other studies. Possibly confounded by high prevalence of risk factors (smoking, immobilization, clotting disorder).
Nota et al. (34)	Retrospective medical records review	EE (pre-2001) and more natural estrogens (post-2001).	2,517 transwomen, median age 30 years, with a mean follow-up duration of 9.07 years.	Standardized incidence ratios of VTE, comparing to reference women, were 5.52 and 3.92 in transwomen initiated on estrogen therapy pre-2001 and post-2001, respectively.	The change in estrogen prescribing practice away from EE therapy led to a substantial decline in incidence of VTE.

Despite that, the current evidence base remains disproportionately influenced by older randomized controlled trials which employed non-physiological estrogens, with little regards for their differential effects. In a recent Cochrane review examining the risk of cardiovascular events in HRT trials, WHI and HERS—both of which employed CEE in intervention arms—accounted for 79%(425/540) of stroke, 88%(312/353) of VTE, and 90%(149/166) of pulmonary embolism events (35). That would inevitably serve to confuse clinicians with a skewed picture of HRT-associated risks being presented. More clarity is certainly needed.

Furthermore, most guidelines on the management of female hypogonadism (e.g., NICE 2017) continue to list COCs as reasonable replacement therapy—with the exception of those for Congenital Hypogonadotropic Hypogonadism, which only recommend E_2_-based HRT (36). In contrast, WHO guidance for the treatment of male hypogonadism has long emphasized that only native testosterone should be prescribed, rather than synthetic androgens. Similarly, HRT prescribing practices in transgender medicine have also evolved over the past 2–3 decades following accumulating data of the significantly lower risk with E_2_ therapy compared to EE/CEE. Another commonality between guidance for androgen replacement in males and E_2_ replacement in trans-females is that it emphasizes the importance of monitoring serum sex hormone with the aim of achieving physiological levels. By contrast, guidance for both young hypogonadal women and older post-menopausal women do not recommend biochemical monitoring (37).

## Data From Transgender Clinical Studies on Comparative Safety of Estrogen Products

For individuals receiving cross-sex hormone treatment, the major goal is to suppress endogenous sex hormone levels and thus reduce biological secondary sexual characteristics, and to replace sex hormone levels consistent with those of the affirmed sex. Importantly, there are no fundamental sex differences in response to sex steroids, and the principles of treatment are very similar to that of HRT in hypogonadal patients. Hence such data are wholly applicable to cis-gender patients.

In 1989, a key publication from a major center in Netherlands on the estrogen treatment outcomes in transwomen reported an alarming 45-fold increase in risk of VTE with EE compared with cisgender controls (31). This finding triggered a change in the treatment protocol to switch patients of age >40 years to transdermal E_2_ in order to lower VTE risk. Although that led to an overall reduction in adverse events, the VTE risk remained high at 20-fold, largely because EE was still being used by a significant proportion of entire cohort, albeit at lower doses (30). It was also concerning that a 3-fold increased risk of cardiovascular mortality was found to be independently associated with long-term users of EE, consistent with the deleterious effects on haemostatic cascade induced by EE (24). Similarly, CEE proved to be greatly unsafe, with an 8-fold increased risk of VTE compared with E_2_ in transwomen seen in a large transgender service in UK (29).

On the other hand, E_2_ demonstrates an excellent safety profile in several transgender studies (Table 1). In a large US cohort of ~700 subjects on E_2_ 4–8 mg/day for a mean duration of 1.9 years, only a single case of VTE occurred (27). Similarly, in a German cohort of 60 subjects on a relatively high oral E_2_ dose of 6 mg/day, including three with underlying thrombotic tendency, only one VTE event was observed (25). Furthermore, in an Austrian study of 162 subjects on transdermal E_2_, no VTE was observed over a median follow-up of 64.2 ± 38.0 months despite a high prevalence of smokers (~60%) and the presence of confirmed thrombophilic disorder (APC resistance) in nearly 10% (26).

EE has been shown to induce APC resistance similar to that of factor V Leiden mutation, as well as increase in plasma protein C and a decrease in plasma protein S, in a dose-dependent manner. Additionally, non-physiological estrogens lead to elevated inflammatory markers such as C-reactive protein and interleukin-6, which could contribute to the prothrombotic milleu (38). Besides first-pass liver effect driving haemostatic dysregulation, higher prothrombotic tendencies are present with other modes of administration (transdermal and transvaginal) as well, providing evidence for a direct pathway induced by the molecular structure (39, 40).

## 17β-estradiol Replacement Facilitates Treatment Individualization

Another major advantage of E_2_ over EE/CEE is the feasibility for dose adjustment according to serum E_2_ concentration. This is important as bioequivalence between different administrative forms (oral tablet, gel, and patch) is not well-established and subject to wide interindividual variation (13). Moreover, titrating to robust physiological E_2_ levels has been correlated with positive outcomes on metabolic parameters and carotid intima media (23, 41). Normative E_2_ values derived from healthy normally menstruating females would serve as a good guide to dosing (14).

## Conclusion

The choice of estrogen formulation is vital to ensure optimisation of safety and treatment efficacy. Compelling data from recent literature supports the use of E_2_ over EE/CEE to avoid the excessive vascular risk that the latter formulations are associated with. Further studies should seek to build on available evidence and provide greater clarity on estrogen replacement to empower clinicians and patients to make better therapeutic decisions.

## Author Contributions

All authors listed have made a substantial, direct and intellectual contribution to the work, and approved it for publication.

### Conflict of Interest

The authors declare that the research was conducted in the absence of any commercial or financial relationships that could be construed as a potential conflict of interest.
